# Risdiplam: therapeutic effects and tolerability in a small cohort of 6 adult type 2 and type 3 SMA patients

**DOI:** 10.1186/s13023-024-03442-0

**Published:** 2024-11-20

**Authors:** Gianmarco Severa, Maria del Carmen Alfaro, Christophe Alimi Ichola, Hussein Shoaito, Sarah Souvannanorath, François-Jerôme Authier, Edoardo Malfatti

**Affiliations:** 1 Reference Center for Neuromuscular Disorders, AP-HP Henri Mondor University Hospital, 1 Rue Gustave Eiffel, Créteil, 94010 France; 2 University Paris Est Créteil, Inserm, U955, IMRB, Créteil, F-94010 France; 3 Instituto Nacional de Ciencias Neurológicas, Lima, Perú; 4 Institut Universitaire de Kinésithérapie, Faculté de Santé, Université Paris Est, Fontainebleau, Ile-de France, France

**Keywords:** SMA, Risdiplam, MFM32, Outcome measure, PROs

## Abstract

**Background:**

Risdiplam is a validated treatment for adult SMA patients, but clear guidelines concerning functional assessment at baseline and during the follow-up are still limited, especially in terms of sensible and validated outcome measures able to capture minimal changes in motor performances induced by therapy. The aim of this work is to describe the effect of Risdiplam on a cohort of 6 adult type 2 and type 3 SMA patients, using Motor Function Measure (MFM32) as a standardized scaleto quantify the motor improvements induced by therapy.

**Results:**

Risdiplam at the dose of 5 mg/daily was administered to a population of 6 (4 F;2 M) type 2 (*N* = 4) and type 3 (*N* = 2), adult SMA patients. Two patients were previously treated by Nusinersen, later suspended due to side effects. At baseline, all patients received a neuromuscular evaluation and a MFM32 assessment. After the beginning of treatment, we evaluated MFM32, patient reported outcomes (PROs), and adverse events over 7–27 months of follow-up. The MFM32 showed an increased score ranging from + 2.16% to + 7.29% in 4 patients. The improvement was maintained overtime, with two patients presenting the longest follow-up period of 24 and 27 months respectively. Subdomain D3 was ameliorated in 66.6% of patients. Two patients previously treated with Nusinersen maintained the pre-Risdiplam scores. The HFMSE was also performed and failed to show significant improvements after treatment. All patients reported subjective ameliorations. The commonest PROs were improvements in breath fatigue, voice’s intelligibility, hand strength and dexterity. Adverse effects were mild and decreased over time.

**Conclusions:**

Risdiplam is a well-tolerated treatment in our cohort of adult type 2 and type 3 SMA patients and resulted in improvement or stabilization in motor functions. MFM32 proved to be sensitive to detect changes induced by therapy. Subjective meaningful improvements were sustained overtime especially in bulbar functions, breath fatigue and distal motor abilities.

## Background

Spinal muscular atrophy (SMA) is an autosomal recessive neuromuscular disorder caused by reduced levels of survival motor neuron (SMN) protein due to homozygous deletions or loss-of-function variants in the *SMN1* gene [1]. A homologous gene, *SMN2*, produces only low levels of functional SMN protein due to alternative splicing of its pre-mRNA that excludes exon 7 from the majority of its transcript [2]. Adult patients actually represent one third of all SMA [3] but clear data regarding natural history, clinical and therapeutic management remain lacking [4]. Nusinersen and Risdiplam are currently available as disease modifying treatment for symptomatic adult 5q-SMA patients only [4]. Risdiplam is able to improve the production of a full-length and functional SMN protein, binding to *SMN2*’s premature mRNA molecule and preventing the removal of exon 7 [5]. Adult patients, especially type 2, often suffer from severe scoliosis making intrathecal administration challenging. Risdiplam (Evrysdi) is approved for adult patients at the daily dose of 5 mg administered orally or via a feeding tube. The advent of disease-modifying therapies led to the need to identify sensible outcome measures able to recognize and quantify minimal changes in motor performance and clinical status due to treatment. This is particularly challenging in adult patients often presenting a low performance-status at baseline [6]. In 2018, the national neuromuscular network, Filnemus, created the SMA multi-disciplinary team meeting (SMDTs) in France, with the aim to homogenize the access to treatment for adult SMA patients in the country [7]. The SMDTs evaluates each case in order to validate the start of therapy, a switch of treatment or a discontinuation, supported by personal experience of the quorum and literature available at the time of the discussion [7]. Motor Function Measurement 32 (MFM32) is a widely used French functional motor scale that has been already used in clinical trials as functional outcome measure in patients treated with disease-modifying therapy, including Risdiplam [8].

In contrast, it has been reported that other scales such as Hammersmith Functional Motor Scale Expanded (HFSME) and Revised Upper Limb Module (RULM) can fail to reflect individual improvement in type 2 and non-ambulant type 3 treated adult patients, due to a floor and/or and ceiling effect [9].

Currently only few data of adult patients treated by Risdiplam are published [10, 11]. The aim of this work is to describe our experience of a small cohort of adult type 2 and type 3 SMA.

## Materials and methods

This is a prospective study including non-ambulant adult 5q-SMA patients followed at the Neuromuscular Reference Center, of Henri Mondor Hospital. Disease onset and progression, distribution of muscle weakness, SMA type, *SNM2* copies were assessed. Informed consent was obtained from all patients in agreement with local ethical committees (DC-2012-1535 and AC-2012-1536) and with the 1964 Helsinki declaration and its later amendments. The methodology and the data privacy policy (method of reference MR-003) were approved by the local Ethics Committee.

Clinical, genetic and functional features of our cohort are summarized in Table 1.

**Table 1:** Clinical and genetic features

Patient	P1	P2	P3	P4	P5	P6
Age at onset (months)	6 m	18 m	24 m	24 m	24 m	18 m
Age (years)	41	41	53	65	22	21
Sex	F	F	F	M	F	M
SMA type	2	2	2	2	3	3
Best motor milestone	SITTER	SITTER	SITTER	SITTER	WALKER (until the age of 2 years)	WALKER (until the age of 10 years)
Motor function status	Non ambulant	Non ambulant	Non ambulant	Non ambulant	Non ambulant	Non ambulant
*SMN2* copy numbers	3	2	3	4	3	4
Prior Nusinersen	no	no	no	no	yes	yes
Risdiplam length of treatment (months)	12 m	24 m	27 m	10 m	11 m	7 m

General features of the population. The length of Risdiplam administration is considered at last clinical and MFM evaluation reported in this work

All patients received Risdiplam at the dose of 5 mg/daily and each treatment indication was approved by the SMDT [7]. Four patients were naive while P5 and P6, two non-ambulant SMA type 3 patients, were previously treated by Nusinersen for a total of five intrathecal administrations respectively, later suspended due to recurrent sciatic pain for P5 and spinal surgery for P6 (Table 2).

**Table 2:** Pre-risdiplam treatment of non-naïve patients

	Number of Nusinersen administrations	Reason of discontinuation
P5	5	Recurrent sciatic pain
P6	5	Spinal surgery

History of treatment in non-naïve patients of the population

At baseline all patients received: (i) a neuromuscular evaluation focusing on residual motor abilities and Medical Research Council (MRC) muscle testing; (ii) motor performance status was evaluated by an expert physiotherapist (CAI) using Motor Function Measurement 32 (MFM32) [12] and Hammersmith Functional Motor Scale Expanded (HFMSE) [13].

After starting Risdiplam at each follow-up time point, these parameters were evaluated: (i) neuromuscular assessment; (ii) MFM32 (iii) HFMSE; (iv) patients reported outcomes (PROs) and side effects. Clinical improvements were evaluated focusing on PROs, which resume motor-functional performances and global ameliorations reported by patients. The mean period of follow-up is 16 months, ranging 7–27 months. MFM32 and HFMSE modifications were calculated comparing the scores before starting Risdiplam and the last assessment under therapy.

### Statistical analysis

The effect of treatment has been evaluated according to MFM32 and HFMSE. Concerning MFM32 the results for each domain (D1, D2 and D3) are expressed as a percentage in relation to the maximum score, and the total value is the sum of all the scores divided by 96 and multiplied by 100, being a score > 3%, a clinically significant improvement [13]. HFMSE contains 33 items that are rated on a scale of 0, 1, 2 with a total achievable score of 66. These items are organized in order to limit changes in position and therefore limits fatigue for the patient. Clinically meaningful ameliorations were defined as a change in the final score of ≥ 3 [14].

## Results

### Population characteristics

This cohort is composed by 6 patients (4 F;2 M) with SMA type 2 (*N* = 4) and SMA type 3 (*N* = 2) aged 21–65 years, with a mean age of 40.5 years (Table 1).

### MFM32 and HFMSE modifications

**Fig. 1:**
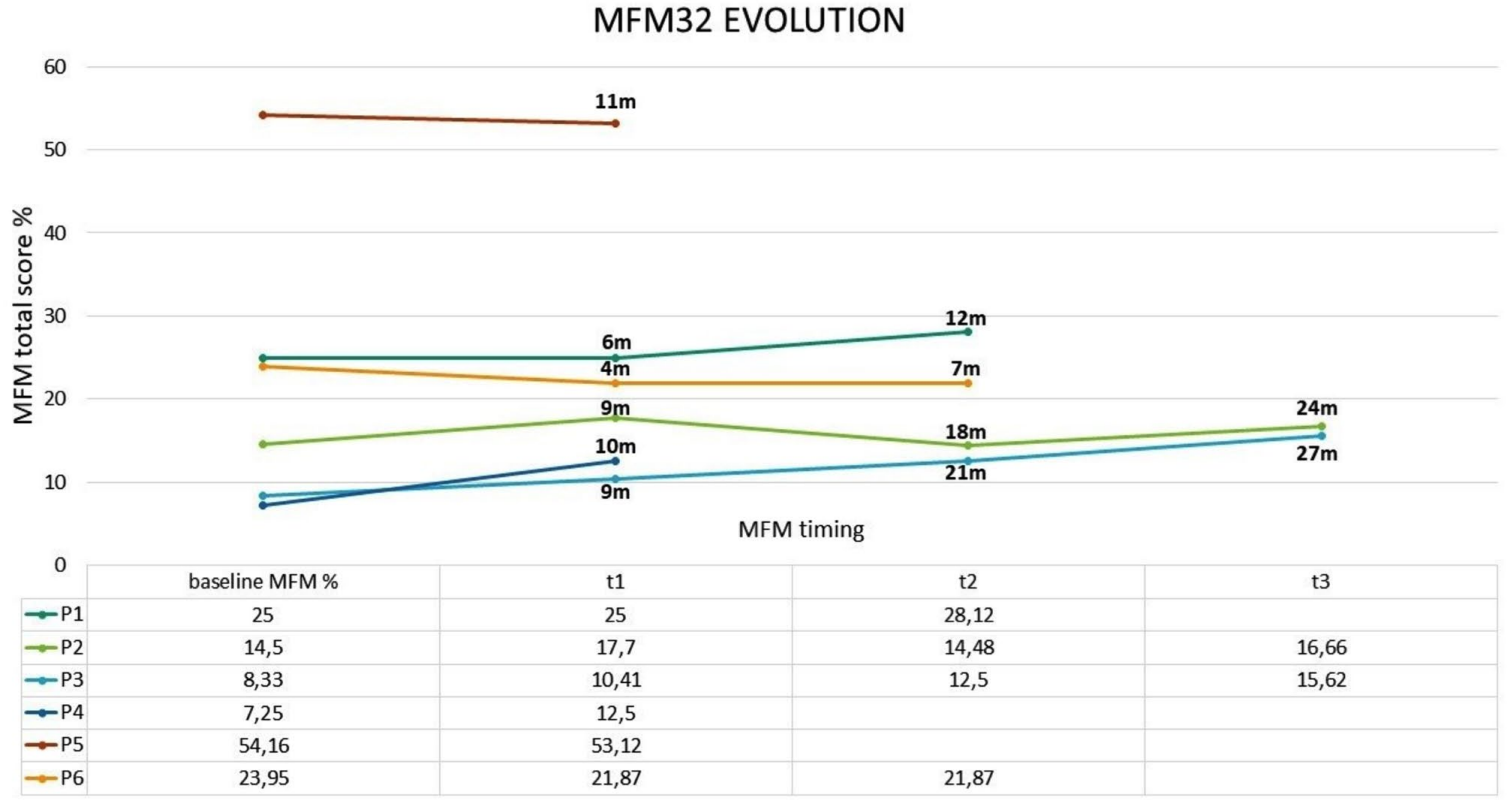
MFM32 global score evolution under Risdiplam. t1, t2 and t3 represent three consecutive time-points. For each patient is indicated in months the duration of treatment at all timepoints

MFM32 and HFMSE total score changes after Risdiplam are reported in Table 3. The evolution of MFM32 final score with all time points for each patient is shown in Fig. 1.

**Table 3:** Changes in MFM32 and HFMSE total score after Risdiplam

Patient	P1	P2	P3	P4	P5	P6
Age (years)	41	41	53	65	22	21
Sex	F	F	F	M	F	M
SMA type	2	2	2	2	3	3
*SMN2* copy numbers	3	2	3	4	3	4
Length of treatment (months)	12 m	24 m	27 m	10 m	11 m	7 m
MFM32 total score before Risdiplam	25%	14.5%	8.33%	7.25%	54.16%	23.93%
MFM32 total score after Risdiplam	28.12%	16.66%	15.62%	12.5%	53.12%	21.87%
ΔMFM32 total score	**+ 3.12%**	+ 2.16%	**+ 7.29%**	**+ 5.25%**	-1.04%	-2.06%
HFMSE total score before Risdiplam	2/66	0/66	0/66	0/66	17/66	0/66
HFMSE total score after Risdiplam	2/66	0/66	2/66	1/66	14/66	0/66
ΔHFMSE total score	0	0	+ 2	+ 1	**-3**	0

ΔMFM32 and ΔHFMSE total score is considered comparing baseline evaluation and the last scale assessment after the start of treatment. Length of treatment is indicated in months for each patient. Significant changes (> 3% for MFM32 and ≥ 3 for HFMSE) are indicated in bold

After a mean period of 16 months of treatment, MFM32 global score showed a clinically significant improvement in three patients (*N* = 3; 50%): P1 = + 3.12%, P3 = + 7.29%, P4 = + 5.25%; while P2 showed an increase of + 2.16%.

P5 and P6, after switching on Risdiplam showed non-significant decrease in final MFM32 score, P5: -1.03% and P6: -2.06%, without changes in their clinical status.

**Fig. 2:**
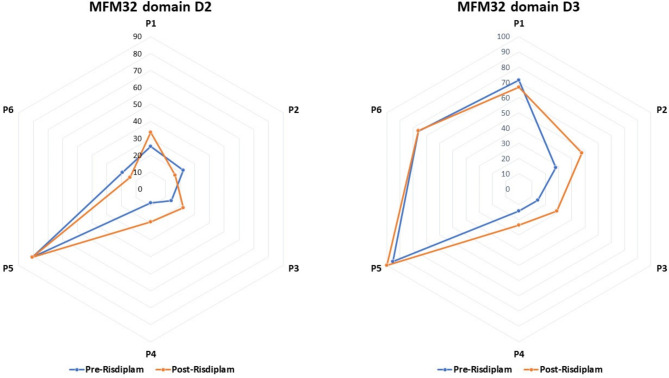
Evaluation of MFM32 domains D2 and D3. D2 (axial and proximal limb function), showed heterogeneous changes after Risdiplam with improvements in half of patients. D3 (distal motor function), the majority of patients revealed ameliorations under treatment. The greatest improvement was observed in patients with lower performance *status* at baseline (P2, P3 and P4). A highest performance at baseline was noted for type 3 patients (P5 and P6)

MFM32 subdomains’ evolution under Risdiplam (Fig. 2) were analyzed comparing baseline and last time point evaluation for each patient.

D1 (standing positions and transfers) showed no clinically significant amelioration after treatment. P1 revealed a non-significant improvement of + 2.56%; P5 presented a decrease of -5.13%. All the other patients showed at baseline D1 = 0 and no modifications were observed after treatment.

D2 (axial muscle and proximal limb function) revealed a clinically significant improvement in three patients (*N* = 3, 50%), P1: +8.33%; P3: +8.42% and P4: +11% respectively. P2 and P6 showed a decrease of -5.33% and − 5.45%. P5 remained stable.

D3 (distal limb motor function) revealed a clinically significant amelioration in four patients (*N* = 4, 66.6%), P2: +19.62%; P3: +14.32; P4: +9.53% and P5: +4.76% respectively. P6 showed a non-significant improvement of + 0.03%. P1 presented a decrease of -4.75%.

HFMSE final score showed no clinically significant amelioration after treatment. Three patients remained stable, while P3 and P4 respectively presented a non-significant improvement on the final score of + 2 and + 1. A clinically significant worsening of -3 in final score was detected for patient P5 similarly to the score showed by MFM32.

### Patient reported outcomes

**Fig. 3:**
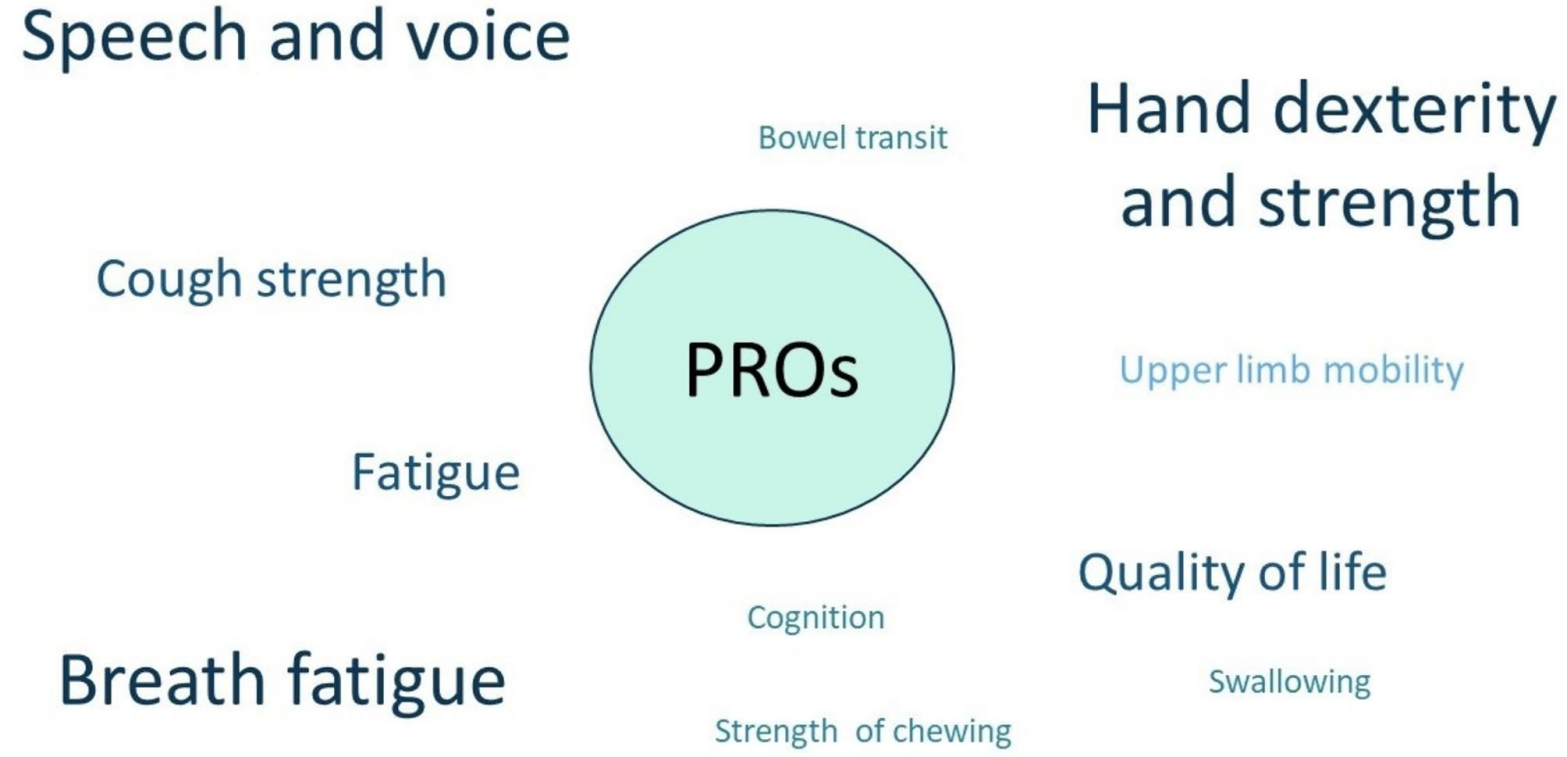
Patient reported outcomes (PROs). PROs are described as specific items for each patient. Font size depends on the frequency of PRO’s amelioration in the cohort

Clinical ameliorations were reported on the following items: strength of chewing, swallowing, speech and voice, cough strength, breath fatigue, bowel transit, upper limb mobility, hand dexterity and strength, cognition, fatigue, quality of life (Fig. 3).

Hand dexterity and strength, breath fatigue and voice intelligibility were the three items improved after treatment in five patients (*N* = 5; 83%); followed by cough strength, fatigue and quality of life in four (*N* = 4; 67%); upper limb mobility in three (*N* = 3; 50%); strength of chewing, swallowing, cognition and bowel transit in two (*N* = 2; 33%).

### Adverse events

In our cohort Risdiplam was generally well tolerated, the most frequent adverse events were photosensitivity (*N* = 4; 67%), diarrhea (*N* = 4; 67%), transient elevation of liver transaminases (*N* = 3; 50%) and nephrolithiasis (*N* = 2; 33%). Other transitory adverse events such as vomiting, cough, headache, and dry skin were reported during the treatment.

## Discussion

The neuromuscular approach toward adult patients with SMA has dramatically changed since the arrival of disease modifying therapies. We moved from a pure observational attitude, where patients usually came to regular follow up mainly for administrative reasons, toward an interventional approach with a legitimate request of patients to access therapies.

In France, SMDTs guides the appropriate choice of treatment for adult SMA patients, and it focuses on the main difficulties concerning this new heterogeneous category of treatable patients [7].

Actually, adult SMA patients have treatment expectations and the neuromuscular reference centers propose an early access to disease modifying therapies in order to stabilize or improve their functional skills. This interventional approach leads to new challenges to solve, especially in terms of a standardized follow-up and the evaluation of the effects induced by therapies. No big data are currently available for treated adult SMA patients, and classical motor or functional scales are mainly conceived for children in which a great improvement induced by therapy is expected [12, 15]. Quantification of minimal changes with motor and functional assessment after treatment in adult SMA can be tricky. This is due to residual motor abilities at the beginning of therapies and the absence of reported large cohorts in the literature [10, 11].

In France MFM32 is the most commonly used scale to assess the functional status of adult patients at baseline and after treatment [7]. MFM32 has proved to be an useful tool able to capture clinical changes induced by Risdiplam in clinical trials for both children and adults type 2 and 3 SMA patients [8].

The MFM32 evaluation after treatment showed a general and sustained improvement in global score in most patients of the cohort. These ameliorations in motor abilities are maintained overtime, and the longest period of follow up was of 24 and 27 months for P2 and P3 respectively.

Focusing on single subdomains, D3 (distal motor skills) presented the highest amelioration in the largest number of patients after treatment, especially in patients with lower performance *status* at baseline. In adult SMA patients, distal motor abilities in the upper limb are more preserved compared to all the others, the clearest response to treatment could be explained by a higher residual motor neurons’ population at baseline susceptible to therapy.

Two patients of the cohort were previously treated by Nusinersen later suspended due to side effects of intrathecal administration, in these patients Risdiplam globally preserved motor improvements acquired. This feature is consistent with results of a recent work on adult patients, in which the treatment with Risdiplam was not associated with a motor worsening in subjects previously treated by Nusinersen [10].

In our cohort HFMSE failed to show clinically meaningful improvements. This is concordant with recent works showing only a HFMSE total score stabilization after Risdiplam treatment [11]. This is probably due to a floor effect in adult SMA patients with few residual motor abilities [9].

Ameliorations in PROs are more evident in bulbar function, respiratory function and distal motor abilities and globally lead to increased autonomy, such as better manipulation of the wheelchair’s joystick, voice intelligibility and quality of life.

The assessment of respiratory function response under treatment, as changes in the percentage of predicted forced vital capacity (FVC%), was not systematically assessed in each time-point and so was not used to evaluate the response to treatment, but in a recent work on adult patients under Risdiplam is confirmed to show sensible changes in percentage of predicted FVC [10]. Risdiplam is a safe and generally well tolerated treatment in adult SMA patients [16]. In our cohort adverse effects were mild and decreased over time.

## Conclusions

Risdiplam is an effective treatment for adult SMA patients. MFM32, showing sustained amelioration overtime in the majority of patients, is confirmed to be a useful tool able to detect minimal changes in motor function induced by the therapy. Improvement in distal motor abilities after treatment was observed in the largest number of patients. Amelioration in bulbar function and respiratory fatigue were also reported by patients. Clinical meaningful improvements were sustained over two years of therapy. Risdiplam is a safe treatment in adult SMA patients, no major adverse effects have been reported.

This study has limitations due to the small size of the population, the heterogeneity of time points of evaluation, and the absence of a placebo control group, raising the possibility of a placebo or a learning effect. Future efforts to conduct clinical studies in larger cohorts are highly desirable in order to better characterize and quantify the effects of Risdiplam on adult patients, and to establish effective outcome measures.

## Data Availability

All main data generated or analyzed during this study are included in this published article. Additional data regarding specific patients clinical features are available and accessible in the following link on reasonable request to the corresponding author in order to protect patients privacy according to the French and European legislation. https://drive.google.com/drive/folders/1SOgIR93usCGDyD_qo4btBEE7HHE4ksx6?usp=sharing.

## Abbreviations

FVC: Predicted forced vital capacity
MFM: Motor function measure
MRC: Medical research council muscle testing
mRNA: Messenger RNA
PROs: Patient reported outcomes
SMA: Spinal muscular atrophy
SMDT: Multidisciplinary team meeting dedicated to adult SMA patients
SMN: Survival motor neuron protein
SMN1: Survival motor neuron 1 gene
SMN2: Survival motor neuron 2 gene
